# Challenges and Future Prospects of Pakistan’s Animal Industry: Economic Potential, Emerging Trends, and Strategic Directions

**DOI:** 10.3390/vetsci12080733

**Published:** 2025-08-04

**Authors:** Ejaz Ali Khan, Muhammad Rizwan, Yuqi Wang, Furqan Munir, Jinlian Hua

**Affiliations:** 1 College of Veterinary Medicine, Shaanxi Centre of Stem Cells Engineering & Technology, Northwest A&F University, Xianyang 712100, China; aliejaz686@yahoo.com (E.A.K.); rizwanmalik4911@gmail.com (M.R.); 17791703611@163.com (Y.W.); 2 College of Veterinary Medicine, Northwest A&F University, Xianyang 712100, China; vetfurqan@gmail.com

**Keywords:** livestock, poultry, fishery, pets, economy, veterinary, animal industry

## Abstract

Livestock, poultry, and fisheries play an important economic role in Pakistan’s animal industry. The pet industry is also emerging and contributing to the country’s economy and people’s emotional well-being. This review provides insight into the current challenges and future directions of the animal industry in Pakistan. Livestock, poultry, and fisheries provide an economically beneficial source of milk, meat, and eggs; however, they face challenges such as disease outbreaks, antimicrobial resistance, climate change, natural disasters, and a lack of proper policies. Likewise, humans benefit from companion animals that provide emotional attachment. Moreover, the pet food market has also shown potential growth, contributing to the country’s economy. Due to the close association between animals and humans, both are at risk for infectious disease transmission. Challenges such as the lack of strong animal welfare laws and the increasing number of stray dogs and cats threaten human safety and that of other animals. We highlight current problems and additional approaches to the management of livestock, poultry, fisheries, and pets, which need to be addressed to further advance the animal industry in Pakistan.

## Simple Summary

Pakistan’s economy relies heavily on livestock, poultry, and fisheries, which produce essential products such as milk, meat, and eggs. The growing pet industry also offers economic benefits and contributes to people’s mental well-being. However, the industry is not lacking in challenges, including disease outbreaks, antimicrobial resistance, climate change, natural disasters, and inadequate policy frameworks. Although providing emotional health benefits as pets, companion animals are also capable of transmitting zoonotic infections. The animal food industry is expanding, contributing significantly to the economy; nevertheless, problems such as the lack of sufficient animal welfare laws and an increasing number of stray animals, which also threaten both animal and human welfare, are on the rise. Comprehensive and authentic policies to address these challenges are essential for the sustainable development and prosperity of the animal industry in Pakistan.

## 1. Introduction

Pakistan’s animal industry is vital to the country’s economy. It supports agriculture, food security, and livelihood [1]. It includes diverse sectors such as livestock, poultry, fishery [2], and companion animals (pets) such as dogs and cats, collectively sustaining a significant workforce in the economy [3]. However, the animal industry in Pakistan faces considerable challenges, such as disease outbreaks, antimicrobial resistance (AMR), climate change, natural disasters, lack of proper policies, and several other challenges, for example, poor management and breeding practices, a shortage of veterinary professionals, and limited access to essential medicines and vaccines [4,5,6,7,8,9].

Agriculture is also critical to the economy in underdeveloped countries such as Pakistan. It is essential to Pakistan’s economic structure [10,11] and contributes approximately 24.04% to the gross domestic product (GDP), with a growth rate of 6.25% annually [12]. Approximately 42.3% of the labor force participates in agriculture, indicating its significance for supporting rural communities and sustaining livelihoods [10,13]. Similarly, the livestock sector is essential for sustainable development and public health. It creates employment opportunities for millions of rural communities and engages labor forces in the Pakistan livestock sector [14]. The livestock industry has shown steady growth, with an increase of 3.89%, indicating stable performance in production [12]. Agriculture’s livestock sector significantly affects the socioeconomic development of rural areas. Pakistan is home to more than 8.5 million individuals, of which 35% derive direct benefits from livestock production [15,16].

Poultry makes up the largest segment of the agro-industrial sector of Pakistan, employing 1.5 million workers with diversified skills [10]. Poultry serves as a key source of protein and energy, turning agricultural waste into nutritious food like eggs and meat [17,18]. It is considered the cheapest animal protein source. Nevertheless, 66% of Pakistan’s population still lacks enough protein [19].

Fisheries also play an essential role in Pakistan’s economy. They contribute 0.31% to the national GDP, support coastal livelihoods, and play a vital role in food security. Fisheries provide alternative protein sources, reducing reliance of coastal communities on other protein sources like mutton, beef, and poultry [12]. Fish is rich in protein, lipids, ash, and micronutrients like vitamins and mineral. It helps prevent various diseases [20], such as bacterial and viral diseases, and provides vitamins and minerals to address deficiencies.

In addition, companion animals (pets) also contribute to the economy in several ways, i.e., marketing pet foods, accessories, and veterinary care services. Pet ownership is rising in Pakistan, with families moving from traditional views to Western inspired trends, and often due to increasingly smaller household sizes [21]. For instance, families adopt the companionship of pet animals such as dogs, cats, and birds to fulfil their emotional needs. Hence, the market for pet animals and products in terms of pet food, veterinary care, and grooming has witnessed steady growth [22]. Overall, the companion animal industry in Pakistan present significant opportunities for growth and development, fueled by changing lifestyles and increasing disposable incomes among the population [21].

In brief, Pakistan’s animal industry displays the potential to grow and can contribute to the country’s economy. However, the current landscape of Pakistan’s animal industry reveals both challenges and opportunities [15,23,24]. We proposed this review to investigate the current problems and prospects of Pakistan’s animal industry, focusing on two main components: the first part emphasizes the current issues faced by the animal industry; in contrast, the second part focuses on the future development of animal programs.

## 2. Service Provision and Structure Dynamics of the Animal Industry in Pakistan

The provision of veterinary services is the provincial government’s responsibility in Pakistan. Each province has its own livestock department, with several directors providing animal health, breed improvement, research, extension, and disease surveillance [4,25].

In Pakistan, animal health professionals are veterinarians, assisted by para-vet staff. The setup includes veterinary hospitals, dispensaries, and clinics providing veterinary care, benefiting the country’s livestock farmers [26]. Each district has a disease diagnostic laboratory and milk testing labs responsible for detecting microbial presence and water adulteration in milk [27]. However, most of these laboratories lack sufficient resources regarding infrastructure and highly qualified personnel. Less than 40% of farmers have access to livestock extension programs, and the quality of the extension services is poorly maintained [25,28].

## 3. A Comprehensive Overview of Pakistan’s Animal Industry

### 3.1. Introduction to Pakistan’s Geography, Population, and Climate

Pakistan is situated in South Asia, bordered by India to the east, Afghanistan and Iran to the west, and China to the north, with a coastline along the Arabian Sea. The varied topography includes plains, plateaus, deserts, and hilly areas such as the Himalayas and Hindu Kush, each influencing agricultural and pastoral operations in distinct ways [29,30]. Pakistan, with a population of over 255 million, ranks as the sixth most populous nation in the world [31,32]. A substantial segment of the population depends directly or indirectly on agriculture and livestock for their livelihoods, particularly in rural regions where animal husbandry is intricately intertwined with local economies and cultures [33].

Pakistan exhibits diverse climatic zones, including dry and semi-arid regions in the south and west, temperate zones in the north, and monsoonal areas of the east [34]. Climate changes affect the distribution and production of livestock, with specific locations supporting cattle and buffalo grazing, while others support poultry or aquaculture. Nonetheless, concerns such as climate change, droughts, floods, and altered weather patterns are progressively affecting animal health and agricultural productivity [35,36].

### 3.2. Livestock (Beef and Dairy)

**Figure 1:**
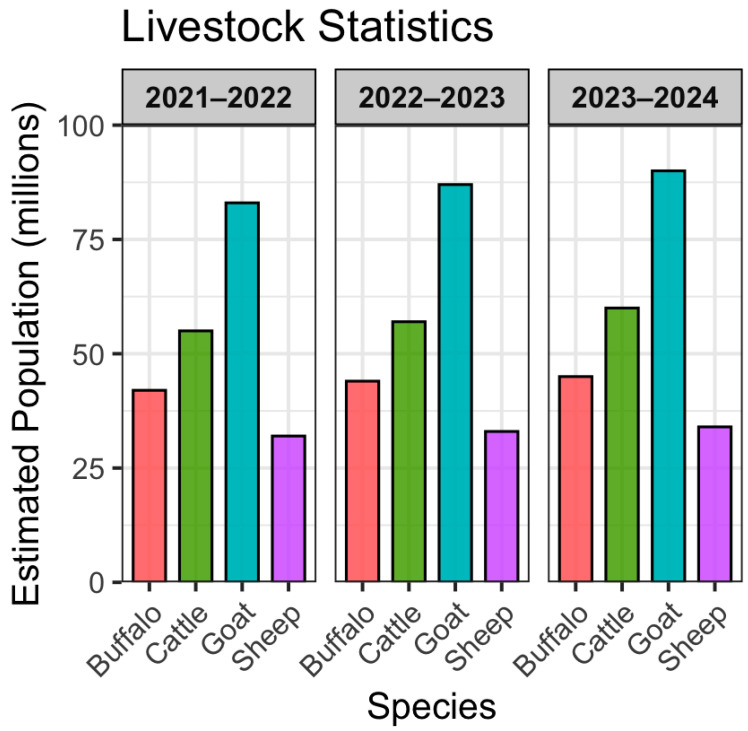
Estimated livestock population of cattle, buffalo, sheep, and goats in Pakistan (R package ggplot2 (v4.4.3) was used for bar chart, in combination with dplyr). Source: Ministry of National Food Security & Research.

Pakistan’s favorable environment supports animal production, with substantial populations of cattle (57.5 M), buffalo (46.3 M), sheep (32.7 M), and goats (87.0 M), as shown in Figure 1 [12,37]. The livestock sector plays a vital role in sustainable development and public health. It could significantly influence national foreign exchange revenues and poverty reduction [10]. Moreover, it creates employment opportunities for millions of people, directly engaging 46% of the labor force and 67% indirectly in the livestock sector [14].

**Figure 2:**
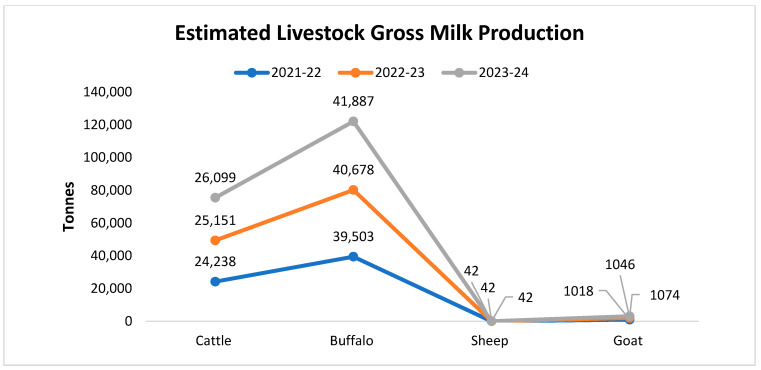
Pakistan’s estimated gross milk production of cattle, buffalo, sheep, and goats. Source: Ministry of National Food Security & Research.

The provincial contribution of milk production of Punjab, Sindh, KPK, and Baluchistan is 63%, 23%, 12%, and 2%, respectively [28]. The milk and meat industry dominate the livestock sector, of which the buffalo (34.6 million) contribute 62% of milk production, while 38% is derived from the cattle population [28], exceeding a combined value up to USD 16.7 million [28,38] per year. Buffaloes as the primary contributors to Pakistan’s milk production, yielding over 41 million tonnes annually significantly more than cattle, sheep, and goats as shown in Figure 2. Milk and meat marketing are considered a quick income source for rural families; in rural smallholder and peri-urban areas, producers own from two to three dairy animals, on average, producing 95% of total milk in Pakistan [38]. Furthermore, about 80% of this milk is sourced from rural areas, with 15% from semi-urban and 5% from urban areas [28,39,40].

**Figure 3:**
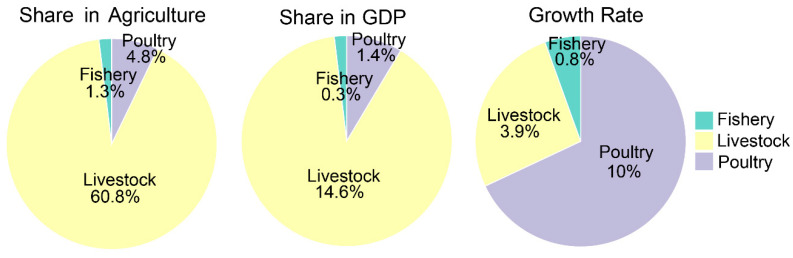
Contribution of Pakistan’s livestock, fishery, and poultry to agriculture, GDP, and growth rate (2023–2024) (R package ggplot2 (v4.4.3) was used for pie chart, in combination with dplyr).

The livestock industry has shown steady growth, with an increase of 3.89%, indicating stable performance in production [12]. Azam et al. [10] reported that in 2015–2016, livestock contributed 58.6% of the gross value added to agriculture and 11.6% of the GDP, showing an increase compared to the previous year’s 56.4% and 11.7%, respectively. The growth was sustained by an approximate 60.84% share of agricultural increase and a 14.64% increase in GDP during 2024, as shown in Figure 3. Similarly, the livestock industry reported an upsurge of USD 20.38 billion in 2023–2024 compared to USD 19.62 billion in 2022–2023 [12]. Additionally, net foreign exchange incomes for this sector generate an expressive contribution of nearly 1.6% to exports. Consequently, the government has evaluated this sector’s development, investigating its intrinsic potential for food security, economic growth, and poverty reduction [41,42]. However, the country’s population upsurge, changing eating behaviors, rise in per capita income, and increasing exports resulted in an increased demand for livestock and its products, such as milk and meat, within the country [10].

### 3.3. Poultry

The poultry sector of Pakistan provides jobs and income for almost 1.5 million people [10,43,44]. Pakistan is ranked as the eleventh largest poultry producer globally, contributing approximately 40.7% to gross meat production (GMP) [45]. The sector comprises over 15,000 poultry farms with a capacity to produce 5000 to 500,000 broilers per year, providing table eggs and poultry meat for human consumption, as shown in Table 1 [45,46], and exhibits an 8–10% growth rate per year, reflecting the inherent potential of this sector [12]. Furthermore, poultry meat accounts for 40–45% of the country’s total meat intake, bringing in USD 6.96 billion per year [10]. Investments in the sector exceed USD 3.71 billion and have recorded an average 7.3% annual growth rate in the past ten years [12].

Poultry provides protein and energy via consuming seven million metric tons of agricultural products and converting waste into edible food such as eggs and meat [17]. In terms of protein sources, the poultry industry covers the gap between the supply and demand for meat protein with the depletion of beef and mutton in Pakistan [19]. Due to the 70% increase in world population and per capita growth of 20% [47], food production has expanded dramatically over the last three to four decades to meet the country’s vast demand. This increase in the population of developing nations increased the per capita demand for food by up to 30%, resulting in a continuous upsurge in hunger and starvation [19]. Similarly, Pakistan lacks enough food—including protein sources—and protein consumption is lower than the standard requirement. For example, the World Health Organization (WHO) recommended that animal protein consumption should be 27 g per day per individual worldwide, while in Pakistan, the value reaches only 17 g per individual per day [48]. Similarly, the per capita availability of poultry meat and eggs in Pakistan is only 5 kg and 51 eggs/year, respectively, compared to 41 kg of meat and 300 eggs [49] annually in developed countries.

### 3.4. Fisheries

Fisheries are also an essential part of Pakistan’s economy, contributing 0.31% to the GDP (Figure 3). They are vital for the livelihood of communities, primarily in coastal areas. Fish meat is one of the alternative protein sources, filling the gap left by the waning dependency on traditional meat sources, i.e., beef, mutton, and poultry in Pakistan. It is rich in macronutrients such as proteins, lipids, ash, vitamins, and minerals. Immunoglobins found in fish meat possess defense mechanisms against various diseases, such as viruses and bacteria [20]. Lipids (long-chain n-3 polyunsaturated fatty acids), alleviate cardiovascular and coronary heart disorders and regulate blood pressure [50]. Vitamin A in fish contributes to proper growth and the development of bones and teeth. Selenium is crucial for the proper functioning of the thyroid gland. Iron plays a vital role in hemoglobin synthesis and helps prevent anemia. Fish naturally contains calcium and vitamin D, which protect against rickets, low bone mineral density, and osteomalacia [20,51].

**Figure 4:**
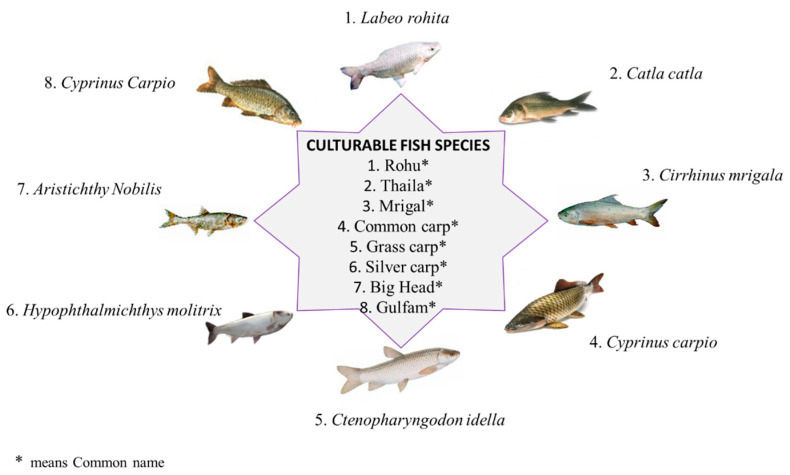
Culturable and edible fish species in Pakistan.

In fiscal year 2024 (July-April), the economic survey reported marine production of 720.9 thousand Mts [12], and the production showed a positive trend in the last decade. Rising international demand fueled the export of fresh and frozen fish and shrimp to the world’s major markets such as Southeast Asia, the Middle East, the European Union, and the U.S. Among these, China, Japan, and Thailand import 206.97 thousand Mts [52]. Hence, exports have shown an encouraging increasing trend of 4.04% [53] compared to the results for 2023. Pakistan has experienced an enormous spike in aquaculture, with freshwater fish cultivation being the most common practice. Rohu, thaila, mrigal [54], common carp, grass carp, silver carp, bighead carp, and gulfam [55] are the popular species cultivated in aquaculture, as shown in Figure 4. However, there is still room for growth and diversity in aquaculture activities, especially in regards to raising commercially useful species such as trout, tilapia, and shrimp. People are largely unaware of the significance of Pakistan’s fisheries, as fish are currently underappreciated and consumed at lower rates than other animal protein sources. Increased fish consumption per capita can improve people’s health in several ways [20], e.g., disease prevention and protein availability. This sector may be further strengthened by generating value-added commodities, deploying modern fishing technology, and improving the fishing community’s socioeconomic situation.

### 3.5. Companion Animals (Pets)

Companion or pet animals are an emerging sector, with a registered population of 190,200 dogs in Pakistan [56]. Five native dog breeds are available in Pakistan, i.e., Bully Kutta, Gull Dong, Gull Terrier, Indian Pariah Dog, and Vikhan [57]. These local dog breeds have evolved with time due to the country’s distinct climate and environment.

Pakistan’s pet industry is experiencing significant growth and transformation, driven by changing cultural attitudes and the rise in the middle-class demographic. Cats are the most popular pets, followed by dogs, birds, and fish. Pet ownership is growing, especially among young individuals (18–24 years old) [58,59]. Pets are becoming more accepted as part of the family, as seen by the rise in the pet accessory businesses and the availability of prepackaged pet food [60]. In Pakistan, 10% of pet owners purchase locally made food, whereas up to 90% rely on imported pet food [58,59]; this is due to the unavailability of local feed products and the lack of quality standards. However, the sector confronts difficulties such as a lack of strong legislation regarding animal care, problems with stray animals, and cultural opposition to particular pets. Additionally, 90% of pet owners visit a veterinarian at least twice a year, and the use of veterinary services is gradually growing [61]. Nevertheless, despite these obstacles, the pet food industry is growing, as seen in the competition of local and foreign companies for consumers’ attention [62].

## 4. Challenges in Animal and Companion Animals Industries

### 4.1. Diseases

Pakistan’s livestock, with a diversified resource potential of 180 million head of cattle, significantly contributes to the country’s economy. In terms of milk production, it produced 61.7 million tonnes of milk and ranked Pakistan the fourth-largest milk-producing country worldwide in 2019 [37]. The inadequate and consubstantial nature of animal vaccines and improper treatment of different animals significantly increase disease risk. Many diseases can be associated with serious animal health issues, leading to economic losses for farmers and health risks for consumers [63]. A proper and continuous vaccination program for foot-and-mouth disease (FMD) is deficient, significantly affecting livestock production.

Lumpy skin disease (LSD) is caused by a virus known as the lumpy skin disease virus (LSDV) [64], which belongs to the family Poxviridae [65] and is one of the most recent economically harmful epidemic diseases in Pakistan. LSD is a threat to the economy due to its effect on the cattle trade, meat production, mortality rate, and its demand for vast volumes of vaccines [66,67,68]. Nizam et al. [69] reported that the primary case of LSDV in Pakistan was recognized in the Jamshoro district of Sindh Province in November 2021. Due to this virus, 36,000 cattle were infected in less than half a year, with a mortality ratio of 0.8%. Hereafter, the Livestock Department declared LSD as an epidemic in the country. The introduction of LSD caused a significant negative economic impact on five million meat vendors and dairy producers. Furthermore, it is reported as a threat to humans through the ingestion of infected animals’ milk and meat [50,70]. Although Pakistan has never experienced an LSD plague, the disease is currently at significant risk of becoming an endemic disease in its neighbors. The disease may be prevented from spreading via a combination of vector management, immunization, rigorous quarantine laws, and limitations on animal movement [65]. Additionally, the animal industry faced significant economic losses during the COVID-19 outbreak [71,72]. Ain et al. [73] reported that the dairy sector suffered significant losses as a result of the COVID-19 restrictions, such as reduced demand and high prices for dairy products.

Coccidiosis is an intestinal parasitic disease caused by the genus *Emeria*, primarily infecting all ruminants, such as cows, buffalo, sheep, and goats [74]. Other major endemic livestock diseases comprise FMD [75], hemorrhagic septicemia [76], Black Quarter [77], and diseases like sheep pox, anthrax, enterotoxaemia, and peste des petits ruminants in sheep and goats [78], which significantly affect the animal industry [23]. Due to the occurrence of so many diseases, poor animal welfare practices, and industry infrastructure limitations, pathogen attacks more easily infect animals, spreading to the whole herd, which drastically affects production.

Pakistan’s poultry industry faces several obstacles, such as disease outbreaks, problems with production costs and quality of feed, and the unavailability of cultivated commodities, such as soybeans, in the market. Avian coccidiosis significantly affects the poultry industry in Pakistan and globally [79]. Similarly, the fisheries are also affected by several diseases categorized as bacterial, parasitic, and fungal [80,81], as shown in Table 2.

In addition, the livestock sector faces feed contamination with mycotoxins such as aflatoxin. The toxins can be transferred from animal feed to milk and can cause potential impacts on health, such as liver cancer, immunologic changes, and growth disorders in children [37]. Aflatoxins, produced by the Aspergillus fungus species, are the most prevalent mycotoxins in various foods and feeds. These toxins are a worldwide problem, particularly in temperate climates where fungi proliferate [91]. More than 20 different derivatives of aflatoxins are present, and their acute exposure contributes significantly to the mortality rate [92,93]. Moreover, 4.5 billion people are affected annually due to prolonged exposure to the toxin, which weakens their immunity [94]. Aflatoxin M1 is the primary metabolite found in milk and is linked to severe health problems [23]. Further consideration of these issues is crucial for maintaining growth and guaranteeing the nation’s food security [12].

### 4.2. Antimicrobial Resistance

Antibiotics treat bacterial infections in livestock, poultry, and fish to promote growth and increase milk and meat production [95]. However, widespread misuse of antimicrobials in these sectors and the inadequate treatment of zoonotic infections have unintentionally contributed to antibiotic resistance [96,97]. Thus, the unchanged portion of the antibiotics escapes into the environment, affecting soil, water bodies, animals, and humans. Due to this spread, alterations develop in microbial sensitivity, leading to antibiotic resistance [98,99].

Milk contamination by antibiotics is a global issue, especially in countries with poor hygiene standards, like Pakistan. Antibiotic residues are significant concentrations of antibiotics or their derivatives found in animal meat or byproducts. These residues contaminate environmental resources, pose direct health risks to humans, and affect dairy production [23,97]. The poultry industry also provides animal protein for human use. However, the public harbors numerous misconceptions about commercial broiler meat and commercial layers’ eggs, e.g., the belief that the overuse of antibiotics in chickens is a stimulus for antibiotic resistance in humans [100].

### 4.3. Climate Change

Risks associated with climate change include the lack of feed, disease outbreaks, water and land resource disruption, declining livestock population, and product quality [63,101]. Pakistan ranks in the top ten countries most impacted by climate change worldwide [102,103], as reported in Germanwatch’s Climate Risk Index. Unstable weather patterns in the country cause both severe floods and severe drought conditions, including glacial outbursts, extreme heat waves, and irregular rainfall. Increased human activity also causes an increase in forest fires, a decrease in migratory species of plants and animals, and fading water sources, such as wells [6]. Consequently, Pakistan’s landscapes and ecosystems are gradually declining. Furthermore, rising sea levels and stronger storms might result in the loss of vital coastal ecosystems like mangroves, which are crucial nurseries for several fish species. Coastal erosion and flooding might also affect fishery habitats. Such severe occurrences are expected to become more frequent and intense due to climate change [8,104], as reported by the Intergovernmental Panel on Climate Change’s (IPCC) Sixth Assessment Report. Hence, these climatic disruptions directly or indirectly affect livestock, poultry, and fisheries and lead to economic losses.

### 4.4. Natural Disaster

Floods mainly affect the agriculture sector. Major crops, which are crucial for livestock, are harmed by flooding, although as a part of the agricultural sector, livestock are a poor source of revenue for satisfying domestic food demands [12,105]. However, floods can affect domestic food, as it is estimated that flooding caused a 37% loss of household food and resulted in 62% of people suffering financial losses; some Pakistanis became refugees due to the devastation of other agricultural sectors, such as forests and fisheries [106]. Azam et al. [10] reported that flooding damaged 40% of the market, resulting in a 55% and 12% loss in agricultural and livestock revenue, respectively, in 2014.

### 4.5. Lack of Suitable Policies

Pakistan’s livestock regulations encourage the horizontal growth of the livestock industry as opposed to the vertical expansion of the sector. There has never been a comprehensive livestock strategy [107]. According to previous literature, it has been reported that the government provided very little funding to support the growth of the livestock industry [108]. The administration has often ignored the livestock industry when announcing agricultural policies. The Livestock Division of the Ministry of Food and Agriculture recommended a livestock breeding program including the cross-breeding of exotic dairy breeds with non-descript indigenous animals [109]. Most policymakers are unaware of the genuine situation and peculiarities of livestock, poultry, and fishery farming [110]. Furthermore, there is a lack of collaboration between the professionals and the dairy farmers due to data gaps, and the issue is exacerbated by inexperienced personnel [111,112]. Farmers are not consulted or trusted when policies are implemented, and their needs are ignored due to a lack of teamwork [110]. In addition, animal welfare legislation is antiquated and fails to align with current scientific and cultural considerations for animal care. Animal welfare standards for farms and zoos may be limited due to inadequate accountability for animal care [113]. Nicole [114] reported in his study that some laws, such as the Prevention of Cruelty to Animals Act (1890), are out of date, and there is no reflection of scientific and cultural understanding of animal welfare. For example, the Cruelty Act (1890) declared that animals feel pain, and the Halal Authority Act (2015) proposes humane treatment and prohibits slaughtering in front of other animals. The implementation of this legislation for animal welfare is lacking [115,116,117]. Therefore, these laws should be regularly updated according to the existing scenarios, and the government should adopt strict policies and amendments to these acts, while ensuring their implementation.

### 4.6. Challenges Faced by Companion Animals (Pets)

Pakistan has a heterogeneous pet culture that faces a myriad of challenges. The lack of strong animal welfare laws allows for the abuse, neglect, and exploitation of animals. It must then be added that the ever-increasing number of stray dogs and cats is a real threat to human health and safety, as well as that of the animals themselves. Pet-related social stigma or discrimination can be influenced by conflicts of religious or cultural beliefs, even among locals [21]. Negligence and mistreatment have been observed in pet owners. Other zoonotic diseases such as rabies, along with the bites of rabid carrier dogs [118], might be a threat to livestock animals, as well as humans.

The pet food market for dogs and cats in Pakistan struggles due to low consumer awareness and a lack of quality products. Many pet owners are unaware of their pets’ nutritional needs and often choose cheaper, low-quality food [59]. Furthermore, the Federal Board of Revenue (FBR) of Pakistan has levied a 50% regulatory tax on the import of dog and cat food. Officials imposed import duties on luxury and non-essential items, including pet food and dairy products, until June of 2025. Import duties on pet food have been raised for the second time, with an increase from 17% to 25% over the levels of the previous year [61], which is a significant challenge for poor pet owners.

Additionally, external forces like natural disasters and pandemics can cause supply chain disruptions, which affect pet care [119]. A previous study by Qureshi et al. [118] reported cases of neglect and mistreatment by pet owners, underscoring the importance of ongoing education and advocacy for the responsible care of pets.

## 5. Future Perspectives and Directions of the Pakistan Animal Industry

### 5.1. Disease Control Measures

Peste des petits ruminants (PPR) is a virus that infects both local and wild ruminants. PPR is presently the focus of global eradication due to its substantial socioeconomic and biodiverse effects in different areas such as Africa, Asia, and the Middle East [16,120]. Different eradication programs such as the National PPR Eradication Program, launched in 2020–2021 with a budget of approximately USD 6.28 million, are devoted to combatting PPR disease in sheep and goats throughout Pakistan, with a commitment to meet international eradication targets by 2030 [121]. The initiative includes essential tasks such as procuring and distributing nearly 20 million doses of PPR vaccine, training for veterinary staff, and providing laboratory equipment. The purpose of these efforts is to position Pakistan as a PPR-free country [12,120], and to be eligible for certification by the World Organization for Animal Health (WOAH).

In addition, the Animal Disease Surveillance and Information System project also secured funding for a specialized surveillance framework (USD 175,724), which means to ensure timely reporting of outbreaks and strengthen response strategies, thereby reducing the economic and public health impacts of animal diseases and ultimately enhancing the nation’s welfare [12].

Moreover, through alliances with organizations, Pakistan is taking significant strides to align with international trade standards such as the WOAH [122,123,124]. This partnership fosters improvements in veterinary infrastructure, enhances the skills of professionals in the field, and supports legislative efforts for animal health and welfare. WOAH also invests in workshops and educational seminars, underscoring its dedication to advancing veterinary services nationwide [125]. Such collaboration can also enhance veterinary service in Pakistan. Establishing some scientific organizations has been pivotal in disseminating scientific research and fostering knowledge within the veterinary community; for example, investment in research education can promote the field of veterinary medicine [126], fostering the prevention of disease and the development of high-quality breeds in terms of milk and meat production. Additionally, some organizations, such as the Pakistan Veterinary Pharmaceuticals Association, may work together for better regulations and innovative solutions to enhance animal industry outcomes nationwide [127]. Moreover, industries should support researchers and invest in research collaboration to further improve livestock, poultry, fishery, and pet animal industries.

### 5.2. Strategies to Combat Antimicrobial Resistance

Antimicrobial resistance in Pakistan has been a persistent concern. Pakistan collaborated with the United Kingdom (UK) for the Fleming Fund Country Grant Phase-I (FFCG), which ran from 2019 until June 2023. The initiative focused on tackling AMR through several crucial activities such as the formulation of surveillance strategies, laboratory renovation, the conducting of comprehensive surveys, and the launch of advocacy campaigns [128,129]. Following the success of the first phase, the UK government has generously allocated an additional grant of GBP 6 million for Phase II of the FFCG program. The second phase, which was inaugurated in January 2024 and will be completed in December of 2025, aims to prioritize collecting high-quality data, performing in-depth analyses, promoting data sharing, and securing long-term investments in AMR solutions [130,131].

In the realm of agriculture, the FAO of Pakistan has made significant strides with different projects such as the development and support of the Pakistan Animal Identification and Traceability System [132,133]. With a budget of USD 231,000, this initiative has successfully developed software modules dedicated to the identification, registration, health monitoring, and traceability of animals. The promising results from the pilot phase have validated the system’s practicality and efficiency, setting the stage for its broader implementation across the country [47,134]. This advancement can facilitate livestock identification and the advancement of Pakistan’s access to the global market.

Additionally, international organizations such as the FAO and WHO are actively working to minimize antibiotic residues in milk. Their efforts also focus on defining standard maximum residual levels (MRLs) for animals and animal products [23], further contributing to public health safeguarding and ensuring food safety.

### 5.3. Climate Change Strategic Plans

Climate change planning, such as addressing livestock management, alleviating heat stress, modernizing farm operations, changing agricultural systems, establishing institutional and regulatory changes, diversification, and expanding Indigenous knowledge are the priorities to be addressed [102]. For this purpose, the government of Pakistan collaborated with the FAO and is being directed by a few government priority areas for 2023–2027, which include climate change and the environment. The FAO is committed to strengthening national frameworks for climate-responsive planning and development through targeted support. It helps to establish and enhance climate information systems at both national and subnational levels to improve governance structures for effective oversight of water and land resources through engaging in the rehabilitation of forestry and rangeland ecosystems, empowering local administrations and communities in disaster-prone areas to better anticipate and respond to climate-related challenges [47,135].

Focusing on the livestock sector, several steps have been made to address the challenges brought on by climate change. This strategy encompasses several key initiatives, such as the National Clean Air Policy (NCAP) and the National Adaptation Plan (NAP), along with various programs to boost climate resilience. Similarly, some entities, such as the national designated authority for international climate funding projects, help to lower carbon emissions and promote sustainable economic development [12]. These initiatives include boosting community resilience and promoting sustainable technologies, which can significantly benefit livestock management. Additionally, the Green Pakistan upscaling program focuses on increasing forest cover, which plays a vital role in enhancing livestock habitats and conserving biodiversity [136,137]. These coordinated actions are crucial for achieving sustainable development and effectively addressing the challenges of climate change in the livestock sector. Furthermore, the FAO supports communities in increasing water use efficiency and promotes the sustainable management of small-scale fisheries and aquaculture [47,136], ensuring their well-maintained habitats.

### 5.4. Natural Disasters Strategies

Plans such as the Livestock Insurance Scheme for Borrowers (LISB) and the Government of Pakistan Markup Subsidy Scheme (GMSS) are designed to assist farmers in the livestock sector, especially in flood-affected areas. These initiatives are implemented by the government to stimulate economic activity in agriculture, specifically focusing on marginalized farmers [49]. For this purpose, the GMSS allocated an impressive sum of USD 35.29 million to benefit approximately 43,465 borrowers. Furthermore, this initiative offers loans, enabling growers and livestock owners to access the necessary funds to improve their operations and productivity. In addition to the GMSS, the Interest-Free Loans and Risk Sharing Scheme for Landless Farmers (IF&RSLF) further assists the farmers and provides support of USD 20.37 million to around 47,425 borrowers [12]. Such financial aid is essential for landless farmers, ensuring they have the resources to cultivate the land and enhance their livelihoods. This dual funding approach shows the government’s dedication to supporting agricultural development and addressing the financial challenges faced by farmers across the country. Together, these initiatives reflect a dedicated effort to support the livestock sector, improve food security, and stabilize the livelihoods of farmers facing difficulties [137].

### 5.5. Policies Enhancement and Implementation

Pakistan’s animal industry lacks a comprehensive policy framework, as previous studies reported that 36% of indigenous livestock breeds were lost due to a lack of conservation efforts and government policies [77]. Moreover, 85–90% of milk remains unprocessed, while 70% fails to meet quality standards, underlining the urgent need for targeted policies to improve breed conservation, milk processing, and food safety regulations [23]. The government could improve livestock, poultry, and fishery divisions by using advanced technology, involving stakeholders, creating a regulatory agenda, and developing policies based on current concerns in the industry by consulting specialists to achieve long-term growth [138]. Furthermore, highly qualified researchers may assist with breeding and other managemental plans. Additionally, proper training and education of farmers can ultimately improve these divisions. Recently, government efforts have supported the One Health concept, which recognizes the connection of environmental, animal, and human health. For example, the COVID-19 pandemic has highlighted the critical need for improved animal health services to reduce global health hazards [72,139]. Furthermore, the Prevention of Cruelty to Animals Act (1890) [116] might be upgraded or amended to reflect contemporary circumstances. Ensuring the adoption of new policies and revisions to the Cruelty and Halal Authority Acts, along with their implementation, is necessary.

### 5.6. Future Prospects of Companion Animals

The government can play an active role in the pet food market to establish quality standards that guarantee nutrition and safety. It can also provide subsidies to local producers and run public campaigns encouraging responsible pet ownership and highlighting the importance of balanced diets and a healthier environment for pets and their owners [58]. Nevertheless, the changing attitude of people toward animals shows improvement and has the potential to raise awareness about animal welfare and protection, reflecting our capacity as humans to empathize not only with each other but also with our fellow beings in the animal kingdom [122].

Furthermore, to address the increased number of dog bite cases in Pakistan, some hospitals have launched initiatives such as Rabies Free Pakistan in Karachi. This program aims to enhance awareness of rabies disease and rabid dogs, as well as to promote vaccination and sterilization of stray dogs in the region. A privately-operated initiative, it demonstrates that mass dog vaccination and effective dog population management can succeed in Pakistan and be replicated throughout the country [138]. Its success depends on the commitment of each district’s municipality to eliminate stray dogs and rabies from their communities. By fostering community involvement and utilizing innovative, low-cost service delivery methods that can be effectively scaled up, we can significantly disrupt the transmission of rabies from free-roaming dogs to people. We must embrace a significant shift in our approach to achieve zero human deaths caused by dog bites by 2030 [140,141,142,143].

### 5.7. The Role of the China–Pakistan Economic Corridor (CPEC) in the Pakistan Animal Industry

Chinese and Pakistani research organizations and trades collaborate regarding animal husbandry and product processing. As part of their agricultural collaboration, they have laid out an extensive National Action Plan for Agriculture Modernization [144]. This partnership encompasses various areas, such as the processing of agricultural products, fisheries, and aquatic products, and the establishment of FMD-free zones in Pakistan [145,146]. Several agreements have been reached to boost Pakistan’s agricultural exports to China; for example, protocols for exporting dairy products, donkey hides, and chilled beef were signed in October 2023 [147]. Additionally, initiatives for creating FMD-free zones and locally producing FMD vaccines are actively being pursued to further enhance the livestock sector.

## 6. Conclusions

Livestock, poultry, and fisheries play an essential economic role in the animal industry of Pakistan. Additionally, the pet industry is emerging and contributing to the country’s economy and people’s emotional well-being. To obtain the maximum benefit from the animal industry, specific challenges, such as disease outbreaks, AMR, climate change, natural disasters, and lack of suitable policies, must be addressed. The Government of Pakistan may take further improvement steps to make this industry one of the most respected industries worldwide.

## Figures and Tables

**Table 1:** The estimated commercial and domestic (meat and eggs) production of poultry.

Estimated Poultry Meat and Egg Production in Pakistan							
	Commercial			Domestic			
	**2021–2022**	**2022–2023**	**2023–2024**	**2021–2022**	**2022–2023**	**2023–2024**	**Units**
Poultry	1632.06	1792.46	1968.71	92.62	94.04	95.5	Million
Eggs	17,944	19,170	20,480	15.78	15.12	14.49	Million
Meat	1846.48	2027.57	2226.54	0.48	0.46	0.44	Tonnes
Layers	68.49	73.28	78.41	-	-	-	Million
Broilers	1548.51	1703.36	1873.69	-	-	-	Million

Source: Ministry of National Food Security & Research.

**Table 2:** Common diseases affect fishery in Pakistan, along with their causes.

S. No	Category	Disease/Predator	Cause	References
1	Bacterial Diseases	Abdominal Dropsy	Bacteria (contaminated water, contact, feed)	[82]
		Fin Rot	Bacterial infection	[83]
2	Parasitic Diseases	Lernaeasis	Ectoparasitic worm (Lernaea sp.)	[84]
3	Fungal Diseases	Saprolegniasis	Fungal infection (Saprolegnia sp.)	[85]
4	Environmental Issues	Anoxia	Oxygen depletion (high temperature, overstocking, biological factors)	[86]
5	Fish Predators	Water Insects (beetles, bugs, scorpions)	Attack fish eggs and fry	[87]
		Amphibians (frogs, toads)	Prey on fry and fish	[88]
		Reptiles (tortoises, snakes)	Eat fish	[89]
		Aquatic Birds (kingfisher, heron, fishing eagle)	Prey on small and big fish	[90]

## Abbreviations

AMR	antimicrobial resistance
GDP	gross domestic product
M	million
GMP	gross meat production
AP	agricultural products
WHO	World Health Organization
Mts	million tons
FMD	foot-and-mouth disease
LSD	lumpy skin disease
LSDV	lumpy skin disease virus
IPCC	Intergovernmental Panel on Climate Change
UK	United Kingdom
FFCG	Fleming Fund Country Grant
FAO	Food and Agriculture Organization
PAITS	Pakistan Animal Identification and Traceability System
FBR	Federal Board of Revenue
PPR	Peste des petits ruminants
WOAH	World Organization for Animal Health
MRLs	maximum residual levels
NCAP	National Clean Air Policy
NAP	National Adaptation Plan
LISB	Livestock Insurance Scheme for Borrowers
GMSS	Pakistan Markup Subsidy Scheme
IF&RSLF	Interest-Free Loans and Risk Sharing Scheme for Landless Farmers
CPEC	China–Pakistan economic corridor
